# Recurrent Episodes of Acute Hiccups With Influenza

**DOI:** 10.7759/cureus.78245

**Published:** 2025-01-30

**Authors:** Lintu Ramachandran

**Affiliations:** 1 Internal Medicine, Javon Bea Hospital, Rockford, USA

**Keywords:** acute hiccups, hiccups, influenza, intractable hiccups, viral infection

## Abstract

Hiccups are a common reflex mechanism and can be classified based on the duration of symptoms. While hiccups are mostly benign and self-limiting, persistent hiccups can cause functional limitations. Hiccups can also be the presenting symptom of an underlying illness. We present the case of a patient who had two distinct episodes of acute hiccups after influenza. We highlight the mechanism behind the hiccups reflex and describe various causes of hiccups, including infections. The varying modalities through which viral infections can lead to hiccups are also described.

## Introduction

Hiccups or singultus is a common transient medical condition that affects almost everyone in their lifetime. Hiccups are caused by involuntary contraction of the diaphragm and intercostal muscles. The involuntary contraction leads to the closure of the glottis, producing the sound associated with hiccups. A large number of causes ranging from central nervous system disorders, lung infections, and myocardial infarctions to medications have been linked with hiccups. We present the case of a patient who had two recurrent episodes of hiccups, a year apart, secondary to influenza.

## Case presentation

A 33-year-old male with no significant past medical history presented to the emergency department with six hours of persistent hiccups. The hiccups had been increasing in frequency since they began, and were happening every 30 seconds, which prompted him to seek medical help. He also reported chills and vomiting that started after the hiccups began. He also reported some myalgias that started after the hiccups as well. He denied any fevers, headaches, shortness of breath, dizziness, runny nose, or any other new-onset symptoms. He had a similar presentation two years prior and tested positive for influenza B at the time. The hiccups at that point resolved with metoclopramide. He did not have any significant past surgical or family history. He was a five-pack-year active smoker. His basic metabolic panel and complete blood count with differential were unremarkable. He had a nasal COVID-19 PCR test done that was negative but tested positive for influenza A. A chest X-ray did not show any active infiltrates or airway obstruction. He was treated with metoclopramide and baclofen, which improved his hiccups. He was discharged home with prescriptions for metoclopramide and oseltamivir for five days. During his primary care follow-up at three days post-discharge, his symptoms had resolved.

## Discussion

Hiccups are primarily classified based on their duration. Hiccups that last less than 48 hours are classified as acute and hiccups lasting over 48 hours are classified as persistent hiccups. Hiccups lasting more than one month are classified as intractable hiccups [1]. Although most acute hiccups are self-limiting, they can hinder the quality of life by interrupting eating, sleeping, and speaking. While most people experience acute hiccups at least once in their lives, the exact prevalence of persistent hiccups and intractable hiccups is unclear. An analysis of published case reports and case-control studies showed a male predominance in persistent and intractable hiccups [2]. Another study conducted in Turkey showed a male predominance in persistent and intractable hiccups associated with gastrointestinal disease but no gender predominance in other causes [3]. Our patient was a young male who presented with two episodes of acute hiccups and was found to have influenza at both times.

Causes of hiccups can be classified as central or non-central. Central causes include strokes, demyelinating diseases, and other injuries to the CNS. In a study conducted on 220 patients with intractable hiccups, 17% of the patients were found to have CNS lesions [4]. Non-central causes commonly include gastroesophageal reflux disease, chest trauma, and mediastinal tumors. A less common cause of hiccups is lung and airway infections. Cases of pneumonia-associated hiccups have been reported in the literature [5-7]. Viral infections, such as herpes simplex virus (HSV), also have been associated with hiccups [8,9]. Recently, there have been several reported cases of COVID-19 cases presenting with hiccups [10-13]. Another reported cause of hiccups is influenza. While there were reported cases of hiccups during the 1910-1930 encephalitis lethargica, also known as epidemic encephalitis, most cases of hiccups were associated with severe symptoms, including altered mental status, and patients had severe flu-like symptoms followed by hiccups [14]. Our patient presented with hiccups followed by myalgias.

The mechanism behind acute hiccups is well-understood. Acute hiccups are caused by involuntary contractions of the diaphragm and an abrupt closure of the glottis. The diaphragm is primarily innervated by the phrenic nerves, which start in the neck on each side, transverse down while interacting closely with soft tissue, neck musculature, blood vessels, the sympathetic chain, and the pericardium, and culminate underneath the diaphragm [15]. As a result, any irritation to the phrenic nerve along the way can lead to acute hiccups. The reflex arc of hiccups is also thought to involve the vagus nerve. Gastroesophageal reflux disease has been shown to induce hiccups and vagal nerve stimulators may be effective in cases with intractable hiccups [16].

Infections such as pneumonia, hepatitis, and pelvic inflammatory disease are thought to cause hiccups from diaphragmatic and phrenic nerve irritation [17,18]. Viral infections, however, have multiple etiologies to cause hiccups. Intractable hiccups in patients with HSV esophagitis are thought to occur from vagus nerve irritation and have been shown to resolve with antiviral treatment [19]. HSV encephalitis can lead to hiccups by affecting the pons [20]. Temporal lobe changes without brain stem involvement have also been associated with hiccups in HSV encephalitis [21,22]. The exact mechanism by which influenza causes hiccups is unclear. There have been two cases of patients who received influenza vaccine and had persistent hiccups. In the first case, a 75-year-old male had severe symptoms, including altered mental status and neurological symptoms from acute disseminated encephalomyelitis, and passed away three weeks after receiving the influenza vaccine [23]. In the second case, the patient had hiccups and neurological symptoms from HSV encephalitis that became reactivated after influenza vaccination [24]. Our patient did not have any neurological symptoms and was never vaccinated against influenza or COVID-19. We hypothesize that repeated coughing during the flu can irritate the phrenic and vagus nerves leading to hiccups. Moreover, myalgias of the upper neck and diaphragmatic muscles may be associated with the hiccup mechanism as well. Our patient, however, did not have any coughing prior to the hiccups but did report some generalized myalgias, which may have contributed to his hiccups.

## Conclusions

In conclusion, while hiccups are mostly benign, they can be the presenting symptom for other underlying illnesses such as influenza. We strongly encourage physicians to look for secondary etiologies in cases with hiccups, especially when they are intrusive or recurrent. Influenza should be on the differential for patients with hiccups, especially in the setting of sick contacts and myalgias.
